# Modulation of Complex-Spike Duration and Probability during Cerebellar Motor Learning in Visually Guided Smooth-Pursuit Eye Movements of Monkeys

**DOI:** 10.1523/ENEURO.0115-17.2017

**Published:** 2017-07-10

**Authors:** Yan Yang, Stephen G. Lisberger

**Affiliations:** 1 State Key Laboratory of Brain and Cognitive Science, Institute of Biophysics, Chinese Academy of Sciences, Beijing, 100101, China; 2 University of Chinese Academy of Sciences, Beijing, 100049, China; 3 Department of Neurobiology, Duke University School of Medicine, Durham, NC 27110

**Keywords:** Floccular complex, long-term depression, neural plasticity, single trial learning, smooth pursuit eye movement

## Abstract

Activation of an inferior olivary neuron powerfully excites Purkinje cells via its climbing fiber input and triggers a characteristic high-frequency burst, known as the complex spike (CS). The theory of cerebellar learning postulates that the CS induces long-lasting depression of the strength of synapses from active parallel fibers onto Purkinje cells, and that synaptic depression leads to changes in behavior. Prior reports showed that a CS on one learning trial is linked to a properly timed depression of simple spikes on the subsequent trial, as well as a learned change in pursuit eye movement. Further, the duration of a CS is a graded instruction for single-trial plasticity and behavioral learning. We now show across multiple learning paradigms that both the probability and duration of CS responses are correlated with the magnitudes of neural and behavioral learning in awake behaving monkeys. When the direction of the instruction for learning repeatedly was in the same direction or alternated directions, the duration and probability of CS responses decreased over a learning block along with the magnitude of trial-over-trial neural learning. When the direction of the instruction was randomized, CS duration, CS probability, and neural and behavioral learning remained stable across time. In contrast to depression, potentiation of simple-spike firing rate for ON-direction learning instructions follows a longer time course and plays a larger role as depression wanes. Computational analysis provides a model that accounts fully for the detailed statistics of a complex set of data.

## Significance Statement

Climbing fiber inputs to the cerebellum appear to have a primary role in cerebellar learning: the presence of a CS is tightly linked to single-trial plasticity, and variation in CS duration affects neural and behavioral learning. Here, we show that the duration and probability of CS responses act as correlated signals to instruct both plasticity in the cerebellum and learning in eye movement behavior in awake behaving monkeys. Modulation of the context of motor errors reveals impressive parallels among the probability of CS responses, the duration of CS responses, neural learning, and behavioral learning. A computational model based on biological measurements reproduces the statistics of a complex set of data.

## Introduction

Climbing fiber inputs to the cerebellum appear to have a primary, yet controversial and nonexclusive, role in cerebellar motor learning (Hansel et al. 2001; Boyden et al. 2004; Carey, 2011; Gao et al. 2012; D’Angelo et al. 2016; Titley and Hansel, 2016). Activation of a neuron in the inferior olive causes a large, long electrical event called a complex spike (CS) in the 5–10 Purkinje cells contacted by the olivary neuron’s axon, called a climbing fiber (Eccles et al. 1967). CSs stand out from the simple spikes caused by mossy fiber inputs because of their irregular, infrequent activity and long-lasting burst with a variable number of spikelets. According to the cerebellar learning theory (Marr, 1969; Albus, 1971; Ito, 1972; Gilbert, 1974) and subsequent experiments (e.g., Ito and Kano, 1982; Linden et al. 1991), climbing fiber inputs cause long-term depression of the parallel fiber to Purkinje cell synapses that are active at the time of the input, and the depression leads to changes in simple-spike firing that cause motor learning. One recent addition to the cerebellar learning theory provides evidence that potentiation in Purkinje cells in the absence of a CS also might contribute to cerebellar motor learning (Schonewille et al. 2010; De Zeeuw and Ten Brinke, 2015; Gutierrez-Castellanos et al. 2017).

The properties of the CS responses recorded in Purkinje cells have a striking correlation with the amounts of both neural and behavioral learning. Under natural learning conditions in behaving animals, the probability of a CS response to a given sensory stimulus varies from ∼0.2 to 0.6 across different modalities and Purkinje cells (Gilbert and Thach, 1977; Ojakangas and Ebner, 1994; Soetedjo and Fuchs, 2006; Medina and Lisberger, 2008). On average, the amount of learning in a given Purkinje cell depends on the probability of a CS response to a given instructive input (Medina and Lisberger, 2008) and the duration of the CS response (Yang and Lisberger, 2014a). These observations raise the possibility that the duration and probability of a CS response to a given stimulus are subject to voluntary modulation (Najafi and Medina, 2013), and that the organism might be able to regulate the potency of climbing fiber–mediated cerebellar learning mechanisms depending on context. Modulation of the synchrony across the climbing fiber inputs to a given area (Llinas et al. 1974, Welsh et al. 1995) also could play a role in motor learning (see Yang and Lisberger, 2013, 2014a).

In the system and cerebellar structure we study, namely smooth-pursuit eye movements and the floccular complex, a CS in response to a learning instruction on one trial is tightly linked to both depression of simple-spike firing rate and behavioral learning on the next trial (Medina and Lisberger, 2008; Yang and Lisberger, 2013; Kimpo et al. 2014; Khilkevich et al. 2016). The absence of a CS is associated with a much smaller increase in simple-spike firing on the next trial. The reliable linkage from CS responses to neural and behavioral learning offers the opportunity both to establish additional correlations between CS responses and neural and behavioral learning and to evaluate in greater detail the properties of CS responses under natural learning conditions.

In this article, we take advantage of our ability to quantify single-trial neural and behavioral learning to show four new features of cerebellar learning. First, modulation of the context of instructive target motions shows new correlative parallels between the probability and duration of CS responses and the resulting neural and behavioral learning. Second, when the system is in a stationary learning condition, neither the probability nor duration of CS responses fluctuates slowly over the course of a learning session. Third, the different time courses of synaptic depression and potentiation in the presence versus absence of a CS response suggest the existence of independent mechanisms. Fourth, comparison of the predictions of computer simulations to analyses of real data constrains a biologically motivated model of trial-over-trial learning that can account for the statistics of a complex set of data.

## Materials and Methods

### Animal preparation

We report data from experiments on two awake, behaving adult male rhesus monkeys. The monkeys were used for recordings from Purkinje cells in the floccular complex during the paradigm invented by Medina et al. (2005) for directional learning in smooth-pursuit eye movements. Different response characteristics of the same population of neurons have been reported in a number of our previous papers (Yang and Lisberger, 2013, 2014a, b). Before the recordings, we implanted a head holder to prevent head motion during experiments, an eye coil to monitor eye position, and a stainless steel recording cylinder to allow access to the floccular complex for single-neuron recordings as detailed in Ramachandran and Lisberger (2005). The surgical procedures used sterile technique, with the monkey under isofluorane anesthesia. Monkeys received opiate or nonsteroidal analgesics for several days after each surgery. Procedures were in accordance with the *National Institutes of Health Guide for the Care and Use of Laboratory Animals* and had been approved in advance by the *Institutional Animal Care and Use Committees* at the University of California, San Francisco, and Duke University.

### Behavioral task

Monkeys were trained to fixate and pursue bright 0.3° or 0.5° spots on a dark background. We presented visual stimuli on a CRT monitor that was placed 30 cm from the monkey’s eye, subtended a visual field of 59° × 47°, and had a refresh rate of 80 Hz. All recordings were acquired in a dimly lit room. After a neuron had been isolated, we presented a baseline block to assess the preferred direction of the simple-spike responses of the Purkinje cell under study. The baseline block comprised ∼80 target motions (called trials), with 10 target motions for 850 ms in each of eight cardinal and oblique directions at a constant speed of 20 deg/s. Target motions followed a standard step-ramp trajectory with a 3° eccentric step to cancel saccades during the initiation of pursuit (Rashbass, 1961). The main experiment delivered four learning blocks that comprised ∼100–400 trials for each Purkinje cell. Monkeys were rewarded with droplets of fluid at the end of each trial if they kept their eye position within an invisible reward window around the position of the target (for details, see Yang and Lisberger, 2013).

### Experimental design

We studied Purkinje cells in the floccular complex that showed strong modulation of simple-spike activity during pursuit eye movements (Stone and Lisberger, 1990a). First, we assessed the direction selectivity of simple spike firing during tracking of step-ramp target motion in the cardinal directions and along the 45° oblique directions. Most Purkinje cells had a strong increase in simple-spike responses for either ipsiversive (toward the side of recording) or downward pursuit and a decrease for contraversive (away from the side of recording) or upward pursuit (Krauzlis and Lisberger, 1996). We defined the on-direction and off-direction for each Purkinje cell according to its simple-spike response. Prior papers have shown that the direction tuning for CS responses in the floccular complex is almost always opposite to the direction turning for simple-spike responses (e.g., Stone and Lisberger, 1990b; Medina and Lisberger, 2008). We verified this and assessed the direction tuning for CS responses during the learning block by computing the probability of a CS in the interval 75–175 ms after an on- versus off-direction instructive change in target direction. Almost all Purkinje cells showed a high probability of CS responses to instructive changes in target direction that took the target in the off-direction for simple-spike firing, and no CS responses to instructive changes in target direction that took the target in the on-direction for simple-spike firing.

We customized the learning target motion for each Purkinje cell to match its direction tuning (Medina and Lisberger, 2008). Consider an example in which the on-direction for simple-spike firing was downward. In a learning trial, the first target motion would comprise a step-ramp with a ramp in a direction orthogonal to the on-direction of the Purkinje cell (e.g., to the right) at a speed of 20 deg/s. After 250 ms of rightward target motion, the target would change direction by superimposing, on the original rightward motion, a 400-ms duration pulse of vertical motion at a speed of 30 deg/s. The instructive target motion could be downward or upward in the on-direction or off-direction for simple-spike responses, to create an ON or OFF learning trial. Our learning trials tell the pursuit system that “rightward motion now means you need to emit a vertical smooth eye movement in 250 ms,” and this is what the system learns. Monkeys were rewarded for their fixation performance at the end of the trial, and fixation contingencies were suspended around the time of the change in target direction to avoid punishing the monkeys for their natural tracking latencies.

We used three different sequences of learning trials that turned out to have different effects on the progression of behavioral learning (Yang and Lisberger, 2010). In the repeated-direction paradigm, we presented the same instruction on 100 consecutive trials, using two different blocks to induce ON versus OFF learning. In the random-order paradigm, the instruction varied randomly from trial to trial between the ON and OFF directions with equal probability for ∼400 learning trials. In the alternating paradigm, we presented >200 trials of instructions in the ON and OFF directions in strict alternation.

### Data acquisition and analysis

To estimate eye position, we measured voltages from a a magnetic search coil system. The signals were passed through an analog differentiator to create voltages proportional to horizontal and vertical eye velocity. The differentiator included a filter that rejected signals at frequencies >25 Hz (–20 dB per decade) and differentiated signals at lower frequencies. We sampled the eye movement signals at 1 kHz on each channel and stored them for offline analysis with single-unit recordings.

To record the activity of single Purkinje cells, we introduced homemade glass-insulated platinum-iridium microelectrodes daily through the previously implanted cylinder and advanced them into the floccular complex of the cerebellum. Purkinje cells showed a high level of spontaneous simple-spike firing interrupted by occasional CS responses at ∼1 CS/s. We amplified extracellular action potentials conventionally, filtered them with a bandpass of 300 Hz to 3 kHz, and digitized the raw traces at 25 kHz for further processing. We used software window discriminators to view the spike train for each trial, identify simple-spike and CS responses, and measure the duration of a CS. We estimated the simple-spike firing rates with a reciprocal interval algorithm (Lisberger and Pavelko, 1986). We counted CS responses in bins with a width of 100 ms, summed across trials that presented identical target motions, and converted the counts to the probability of a CS in each bin. We also measured the duration of each CS, with the experimenter blind to the eye velocity and simple-spike firing rate at the time and to context of the CS and the details of the learning trial (Yang and Lisberger, 2014a).

Most of our data analyses involved measuring how simple-spike firing or eye velocity changed between two consecutive learning trials. We call the two consecutive trials “instruction” and “test” trials, and we define the pair as ON or OFF learning according to the direction of the added target motion in the instruction trial. To assess trial-over-trial changes in firing or eye velocity, we computed the firing rate (or eye velocity) on the test trial minus that on the instruction trial for each millisecond. In some analyses, we also assessed the actual firing rate on test trials minus the baseline firing rate in prelearning control trials. In both cases, we quantified learning based on the mean values across specific brief time windows from 100 ms before to 50 ms after the time of the instructive change in target direction. The choice of an analysis interval that ends 50 ms after the instructive change in target direction ensures that our measures of learning precede any visual feedback and therefore are related to the instruction in the prior trial, rather than to that on the current trial.

### Model of simple-spike learning in PCs

We simulated a series of OFF learning trials for each of 1000 model Purkinje cells. Although our goal was to model the single-trial learning in the random direction paradigm, we chose to simulate only OFF learning trials because the model had no memory beyond a single trial. Thus, there was no need to establish stationary learning conditions by including the ON learning trials. We did, however, include the small potentiation of simple-spike firing that occurs on a test trial after an OFF learning trial that does not evoke a CS response [see Eq. (5)].

For each trial in each model Purkinje cell, we drew a random number to serve as the seed for deciding whether a CS would occur and what its duration would be:

(1) p=N(0.2)

where *N*(*b*) signifies a normal distribution with a mean of zero and a standard deviation of *b*. This distribution renders the occurrence of CS responses stochastic but does not allow the underling probability to vary from trial to trial. To accomplish the latter goal, we drew a random threshold:

(2) t=0.8*U(0,1)

where *U*(*a*,*b*) signifies a uniform distribution between *a* and *b*. We placed a CS on each model trial if *p > t*. The two steps summarized by Eqs. (1) and (2) create a situation in which the probability of a CS varies from trial to trial because the threshold for evoking a CS (*t*) varies.

Next, we established a value of duration *d* for each CS that is related to its probability *p*:

(3) $$d=8*\left[r*p+\sqrt{\left(1-r^{2}\right)}*N\left(0.2\right)+1\right]$$

The value of *r* establishes how strongly the value of *d* is related to the value of *p* and therefore determines the underlying correlation between the probability and duration of CS responses. The scaling factor of 8 in Eq. (3) was determined empirically so that the distribution of CS durations would match the distribution in our data. Finally, for the purposes of numerical simulation, we normalized the duration to yield values mostly between 0 and 2 with a mean of 1:

(4) $$D=\frac{d}{4}-1$$

We will show that analyzing the model data in bins of 10 trials failed to reveal much correlation between CS probability and duration, even when we had intentionally correlated CS probability and duration on single trials. We intuited that we could increase the mean correlation, something we might have to do to reproduce our data, by allowing CS duration to be correlated among trials that occur close to each other in time, i.e., to have temporal correlations. Therefore, we devised a computational procedure that would allow us to control the correlation between CS duration and probability on single trials completely independently of the degree of temporal correlation in CS duration.

After creating the trials and a specific correlation between CS probability and duration using Eqs. (1)–(4), we created maximal temporal correlations by sorting the trials by CS duration. This did not change the underlying correlation between CS duration and probability, but it did create strong correlations between CS probability and duration when analyzed in bins of 10 trials. Then, we disrupted the temporal correlation in a systematic way by shuffling the trials to some degree, swapping the order of the trials for randomly chosen pairs. If we performed zero swaps, we defined the temporal correlation as a value of 1. If we performed 0.8 swaps per trial in the model data set, or 160 swaps when the model dataset contained 200 trials, we defined the temporal correlation as (1 – 0.8) or 0.2.

We modeled the trial-over-trial change in simple-spike firing from trial to trial as

$$\Delta SS_{i,i+1}|CS_{i}=-5.5*D+N\left(14\right)$$

(5) $$\Delta SS_{i,i+1}|∼CS_{i}=1.7+N\left(14\right)$$

where Δ*SS_i_*_,_*_i_*_+1_ is trial-over-trial change in simple-spike firing rate between the *i*th and *i*+1th trials, *N*(*b*) is a normal distribution defined above, and *D* is the normalized duration of the CS. These distributions were chosen to allow the trial-over-trial change in simple-spike firing to match our observed distributions, and we will show in Results that our approach accomplishes this goal. Our Matlab code is available on request.

## Results

### Learning task and properties of single-trial learning

**Figure 1.**
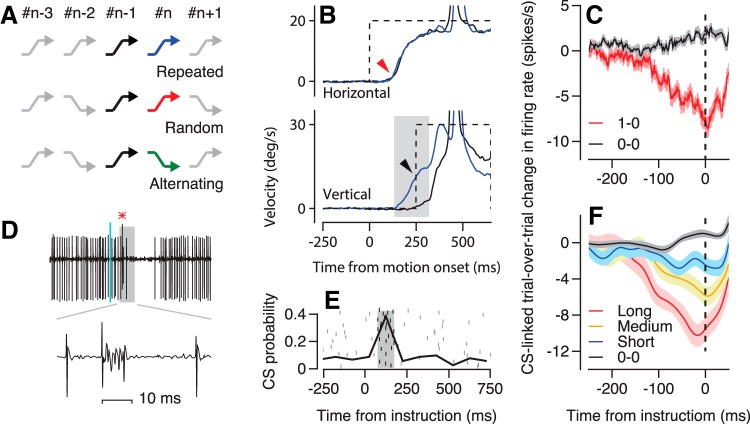
Approaches used to study learning in the direction of smooth pursuit eye movements. ***A***, From top to bottom, the zigzags indicate the trajectories of the learning target motions in the repeated-direction, random-order, and alternating paradigms. ***B***, The superimposed traces show horizontal and vertical velocity as a function of time from the onset of target motion in example trials from one learning block. Dashed and solid traces show target and eye movement. Black and blue traces show responses in the first versus 100th off-direction learning trials in the repeated-direction paradigm. The red and black arrowheads on the velocity records point out the onset of the initial pursuit and the peak of the learned response in eye movements. The gray shading shows the analysis interval for learning. ***C***, Red and black traces show the trial-over-trial change in firing rate versus time for pairs of trials with versus without a CS in the instruction trial. Vertical dashed line shows the time of the instructive change in target direction. ***C*** is reprinted with permission from Yang and Lisberger (2013). ***D***, Simple-spike firing and CS of a representative Purkinje cell in an off-direction learning trial. The red asterisk indicates a CS. ***E***, Raster shows the occurrence of CS responses in relation to the time of the instruction. Black curve shows the probability of CS responses in 100 ms bins. Gray shading shows the analysis interval for CS responses. ***F***, Different color traces show the trial-over-trial change in firing rate versus time for pairs of trials with different durations of CSs in the instruction trial. Vertical dashed line shows the time of the instruction. ***F*** is reprinted with permission from Yang and Lisberger (2014a).

We report on the responses of 34 Purkinje cells that we tested fully through three blocks of direction learning in pursuit eye movements (Medina et al. 2005), including random and alternating blocks (Yang and Lisberger, 2010) of >200 trials each and a repeated block of >100 trials (Fig. 1A). During trials that cause direction learning, eye movements follow the trajectories illustrated in Fig. 1*B*. Approximately 80 ms after the onset of target motion, horizontal eye velocity begins to track the rightward motion of the pursuit target (red arrowhead). Early in a learning session (black traces), vertical eye velocity is mainly reactive to the instructive vertical target motion and remains close to zero until at least 50 ms after the onset of upward target velocity. After 100 repetitions of the same instructive trial in the repeated paradigm (blue traces), vertical eye velocity anticipates the upward target motion and shows a learned response that reaches around 12 deg/s in this stellar example by the time the target starts to move upward (black arrowhead).

For OFF-direction learning trials (instruction in a Purkinje cell’s off-direction for simple-spike responses), the instruction causes an increase in the probability of CS responses in the interval 75–175 ms after the instruction (Fig. 1*E*). In this example Purkinje cell, the probability of a CS response during the analysis interval was 0.4 in the random-direction learning paradigm. Following the criteria established in prior studies (Medina and Lisberger, 2008; Yang and Lisberger, 2014b), we characterized this Purkinje cell as “CS-frequent” because the probability of a CS in the analysis interval was >0.3. We studied only CS-frequent Purkinje cells because they (1) form the overwhelming majority of pursuit-responsive Purkinje cells, (2) have high probabilities of CS responses to an instructive change in target direction, and (3) show the expected reciprocal learned changes in simple-spike firing rate after 100 trials of learning with on- versus off-direction instructions (Medina and Lisberger, 2008; Yang and Lisberger, 2014b). We have not analyzed a small group of CS-infrequent Purkinje cells that had a low probability of CS responses to the instructive change in target direction. Previous articles have shown that CS-infrequent Purkinje cells have wrong-way learned increases in simple-spike firing after 100 off-direction learning trials in the repeated paradigm (Medina and Lisberger, 2008; Yang and Lisberger, 2014b). They are encountered only infrequently, so our sample was not large enough to analyze them in a meaningful way.

Our previous work (Yang and Lisberger, 2013, 2014a) established an approach for evaluating single-trial plasticity contingent on a climbing-fiber input to individual Purkinje cells. We assembled all trials from a random-direction learning block into pairs of consecutive trials. We defined the first and second trial of each pair as the instruction and test trials and selected all pairs with an off-direction instruction on the instruction trial. Then, we divided these pairs into two groups according to whether the off-direction instruction evoked a CS response. We computed the millisecond-by-millisecond difference between the simple-spike firing in the test and instruction trial and averaged across all pairs of trials within each of the two groups. We performed the same analysis for groups of trial pairs defined according to the duration of the CS response in the instruction trial.

As we have published before (Medina and Lisberger, 2008; Yang and Lisberger, 2013, 2014a), single-trial plasticity of simple-spike firing rate is contingent on the presence and duration of the CS on the instruction trial. Comparison of the red and black traces in Fig. 1*C* illustrates that the occurrence of a CS on one off-direction learning trial is linked to a properly-timed depression of simple-spike firing on the subsequent trial (Medina and Lisberger, 2008; Yang and Lisberger, 2013), whereas the absence of a CS is linked to a slight potentiation of simple-spike firing on the subsequent trial. Fig. 1*F* is a reminder that the magnitude of plasticity in simple-spike firing rate depends on the duration of the CS response in the instruction trial. Longer-duration CS responses in Purkinje cells on the instruction trial cause larger plasticity and stronger learning on the test trial (Yang and Lisberger, 2014a). With this background, we now turn to our new results.

### CS properties and single-trial learning and plasticity in different learning paradigms

The correlations between the existence and duration of a CS response and the single-trial plasticity persist across three different learning paradigms that introduce instruction trials in different orders. The details of the time course of learning differed across paradigms, but the probability and duration of CS responses predicted both simple-spike plasticity and behavioral learning in the repeated, random, and alternating learning paradigms. Our prior article showed a strong correlation between the number of spikelets in a CS and its duration (Yang and Lisberger, 2014a), so the analyses presented below would hold as well for the number of spikelets as they do for duration.

As before, we broke each sequence of learning trials into pairs, and we analyzed the pairs that delivered an off-direction learning target motion in the instruction trial. We divided these pairs into sequential groups of 10, and in each group we measured both the average absolute eye velocity in the analysis interval (gray shading in Fig. 1*B*) and the average trial-over-trial change in eye velocity between the instruction and test trials. We analyzed only pairs of trials with an off-direction instruction, and we averaged across groups of 10 consecutive pairs with off-direction instructions.

The learning curve for the trial-over-trial change in eye velocity depended on the sequence of instructions.

**Figure 2.**
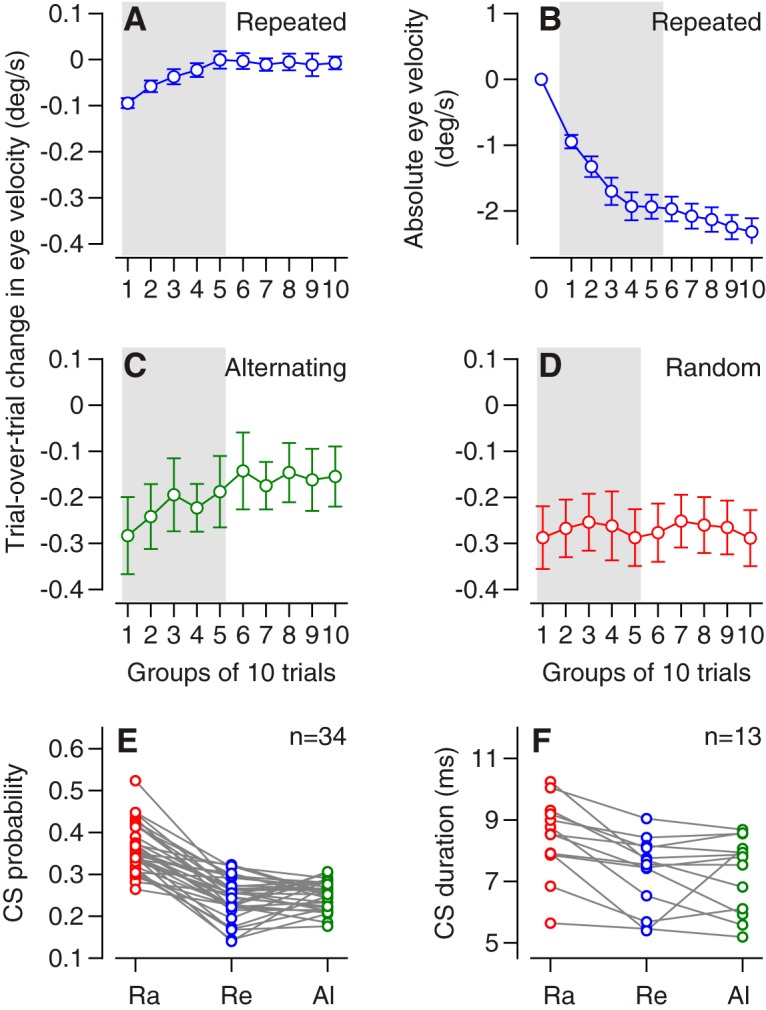
Trial-over-trial learning and properties of CS responses to the instruction with different sequences of learning tasks. ***A***, ***C***, ***D***, Time course of trial-over-trial changes in eye velocity in the analysis interval for learning in blocks of 10 trials. Different graphs show data for repeated (***A***), alternating (***C***), and random-direction (***D***) paradigms. ***B***, Time course of absolute eye velocity in the analysis interval for learning in the repeated paradigm, again in blocks of 10 trials. ***E***, ***F***, The probability (***E***) and duration (***F***) of a CS response to an instruction as a function of learning paradigm. The sets of three connected symbols present data for different Purkinje cells tested in all three learning paradigms. Ra, random; Re, repeated; Al, alternating paradigm. After Bonferroni correction: *p*(Ra, Re) = 1.75 × 10^–12^ and *p*(Ra, Al) = 1.1 × 10^–10^ for probability; *p*(Ra, Re) = 1.45 × 10^–3^ and *p*(Ra, Al) = 0.02 for duration.

In the repeated direction paradigm, the absolute learned eye velocity reached an asymptote within 40 trials (Fig. 2*B*), as we have shown before. We show the absolute learning curve for this paradigm to aid readers in appreciating the details of the trial-over-trial behavioral learning (Fig. 2*A*). As the absolute learning curve saturated, the trial-over-trial behavioral learning declined to close to zero. We regard the saturation after 40 trials as a ceiling effect that probably reflects the limits of early components of pursuit learning. Note that the learned eye velocity of 2.3 deg/s in group 10 in Fig. 2*B* is considerably smaller than the eye velocity of ∼12 deg/s at the time of the instruction in the single stellar example trial of Fig. 1*B*. The smaller averages in Fig. 2*B* result from three factors: (1) averaging across the interval from 100 ms before to 50 ms after the onset of the instruction instead of picking the peak eye velocity, (2) averaging across 10 trials in each group and across 34 neurons, and (3) the huge size of behavioral learning in Fig. 1*D* in what might be the best single-trial example in the entire data set.

In the alternating direction paradigm, trial-over-trial learning was strong early in the learning block and declined to a nonzero asymptote later in the block (Fig. 2*C*). Given that alternation of instruction direction prevented persistent behavioral learning, we have omitted the learning curves for absolute eye velocity. The negative values of trial-over-trial learning indicate that the change in eye velocity was in the direction of the instruction. Thus, unlike humans who show learned eye movements in the direction of the test trial in the alternating direction paradigm (Yang and Lisberger, 2010), monkeys showed learned eye movements in the direction of the instruction trial. We conclude that the monkey pursuit system does not have access to the monkey’s potential cognitive knowledge that the instruction directions would alternate throughout the block of trials. Given that the direction of the instruction alternates, the difference between trials *n* – 1 and *n* will be equal, on average, to the difference between trials *n* and *n* + 1. Thus, the trial-over-trial change in eye velocity for on-direction instructions was almost identical to that for off-direction instructions, but with a positive sign.

In the random direction paradigm, trial-over-trial learning was quite strong throughout the learning block (Fig. 2*D*). The negative values of trial-over-trial learning indicate that the change in learned eye velocity between the instruction and test trials was in the direction of the instructive target motion in the instruction trial. Again, we show only the data for OFF-direction learning and we omit the curves for absolute eye velocity because the randomized directions of the instructive target motions precluded persistent behavioral learning. The learning curve for on-direction instructions was very similar, but positive.

The probability and duration of CS responses to the instructive change in target direction differed among learning conditions, when averaged across the entire learning block. The probability of a CS was higher and the duration of CS waveforms was longer when instructions were presented in a random order versus in repeated or alternating sequences. In the random, repeated, and alternating learning paradigms, the probability of a CS, across all the OFF-direction trials in a learning block, averaged 0.36, 0.24, and 0.25; the duration of CS waveforms averaged 8.44, 7.27, and 7.28 ms (Figs. 2*E*, *F*). These values are lower than the peak values shown in the next figure because they were averaged across the entire learning block. Paired *t* tests with Bonferroni correction revealed highly significant differences between the values for the random and repeated paradigms and the random and alternating paradigms for both duration and probability, and no difference between the values for the repeated and alternating paradigms. *P*-values appear in the figure legend. Note that only 13 Purkinje cells are included in Fig. 2*F* because that was the size of the sample in which isolation remained good enough that we were confident measuring CS duration across all three learning paradigms.

### Behavioral and neural learning curves in different learning paradigms

We consider the relationship between the behavioral and neural learning curves in two steps.

**Figure 3.**
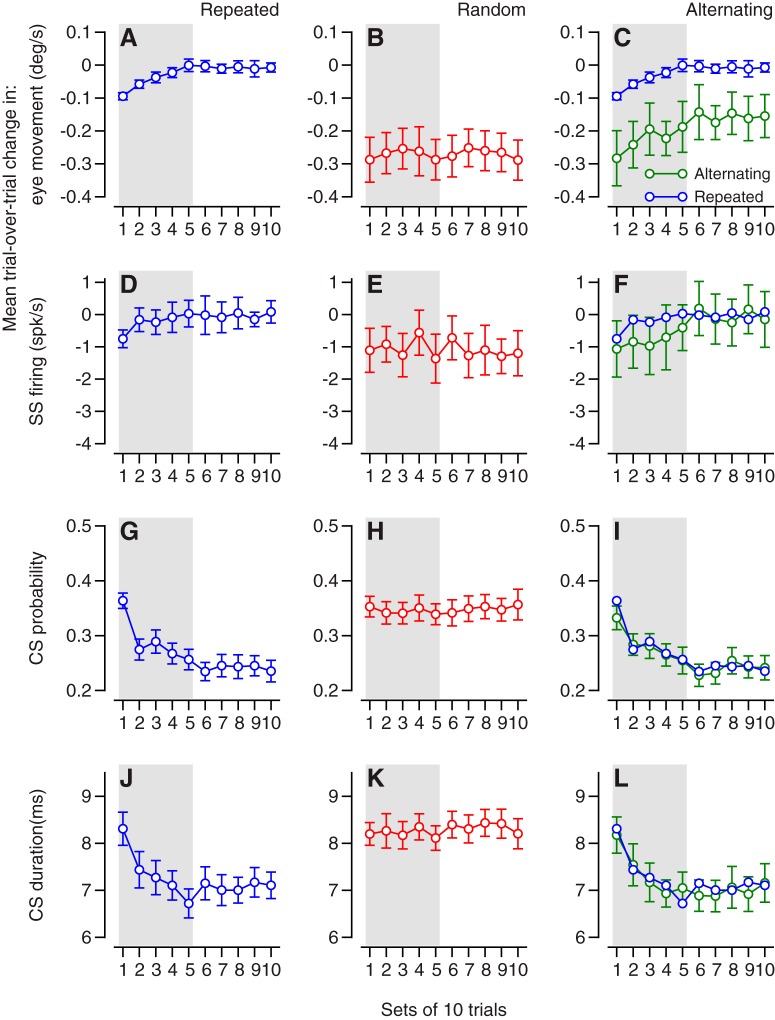
Time courses of pursuit learning in eye movements, trial-over-trial depression of simple-spike firing, CS probability, and CS duration when instructions are present in repeated, random-order, or alternating sequences. From left to right, the three columns summarize the data for the repeated, random, and alternative sequences. Each graph shows a learning curve for data analyzed in bins of 10 trials. ***A–C***, Trial-over-trial changes in eye velocity. ***D–F***, Trial-over-trial changes in simple-spike firing of Purkinje cells. ***G–I***, Probability of CS responses. ***J–L***, Duration of CS responses. Error bars: ± 1SE of the mean. Blue symbols in right column repeat those in left column.

First, we look at the data for each learning paradigm separately. To make it easier to view the relationship between neural and behavioral learning, Fig. 3 repeats the behavioral learning curves from Fig. 2. The trial-over-trial learning curves follow different trajectories in different learning paradigms, but the curves for behavioral and neural learning are correlated within each paradigm. The time courses of duration and probability of CS responses align with the learning curves for simple-spike firing rate and eye velocity (Fig. 3), suggesting some degree of cause and effect.

In the repeated paradigm (left column of Fig. 3), the trial-over-trial changes in eye velocity and simple-spike firing rate were nonzero in the first 40 trials of the block, but then declined to near zero as the absolute learned eye velocity and firing rate reached plateaus. Over the same time course, the probability and duration of CS responses decreased from initial values of 0.38 and 8.3 ms to asymptotes of 0.2 and 7 ms. Bin-by-bin correlation analysis of the averages in Figs. 3*A* and *D* yielded *r* = 0.91. The more telling bin-by-bin analysis on the data for each individual Purkinje cell yielded values of *r* that averaged 0.13. This value seems like a poor correlation, but it is impressive in the context of the variance of 200 (spikes/s)^2^ in the trial-by-trial change in simple-spike firing rate for single trials, which reduces to 20 (spikes/s)^2^ for bins of 10 trials. Simulations based on adding noise with a variance of 20 (spike/s)^2^ to the averages in Fig. 3*D* yielded values of *r* less than 0.13. We conclude that the trial-over-trial change in firing rate tracks the trial-over-trial change in eye velocity well in the repeated-direction paradigm, within the bounds of the noise in Purkinje cell simple-spike firing rate. However, the degree of variation across single trials implies that downstream neurons will have to pool the activity of many Purkinje cells to take advantage of the parallels between firing rate and eye velocity.

In the random-direction paradigm (middle column of Fig. 3), the trial-over-trial changes in eye velocity and simple-spike firing rate were steady across the entire learning block, as were the probability and duration of CS responses.

In the alternating-direction paradigm (right column of Fig. 3), trial-over-trial simple-spike firing and CS probability and duration were very similar to those in the repeated paradigm (compare blue and green symbols). However, the trial-over-trial changes in eye velocity were quite different between the two paradigms (see explanation below). Again, bin-by-bin correlation analysis of the averages in Fig. 3*C* and *F* yielded *r* = 0.85. The more telling bin-by-bin analysis on the data for each individual Purkinje cell yielded values of *r* that averaged 0.15. As before, the low value can be attributed to the noise in simple-spike firing rate, and we conclude that firing rate tracks the trial-over-trial change in eye velocity well in the alternating-direction paradigm.

Second, we compare the data across learning paradigms. The declines in CS duration and probability for the repeated and alternating conditions were almost identical (Fig. 3*I*, *L*). The declines in simple-spike plasticity also agreed quite well for the repeated and alternating paradigms (Fig. 3*F*), with somewhat more plasticity early in a block of alternating-direction learning. There is, however, a striking difference between the amplitudes (but not the time courses) of single-trial eye velocity learning in the alternating-direction paradigm versus the repeated-direction paradigm (Fig. 3*C*). We think the amplitude of the eye velocity learning is inflated artificially in the alternating-direction paradigm because of the consistent superposition of two contributions to eye velocity in the same direction: decay of the single-trial learning that was present in the prior trial and the actual learning that appears in the current trial. In contrast, the decay and acquisition of single-trial learning compete with each other in the repeated paradigm, potentially underestimating the magnitude of single-trial plasticity and learning.

**Figure 4.**
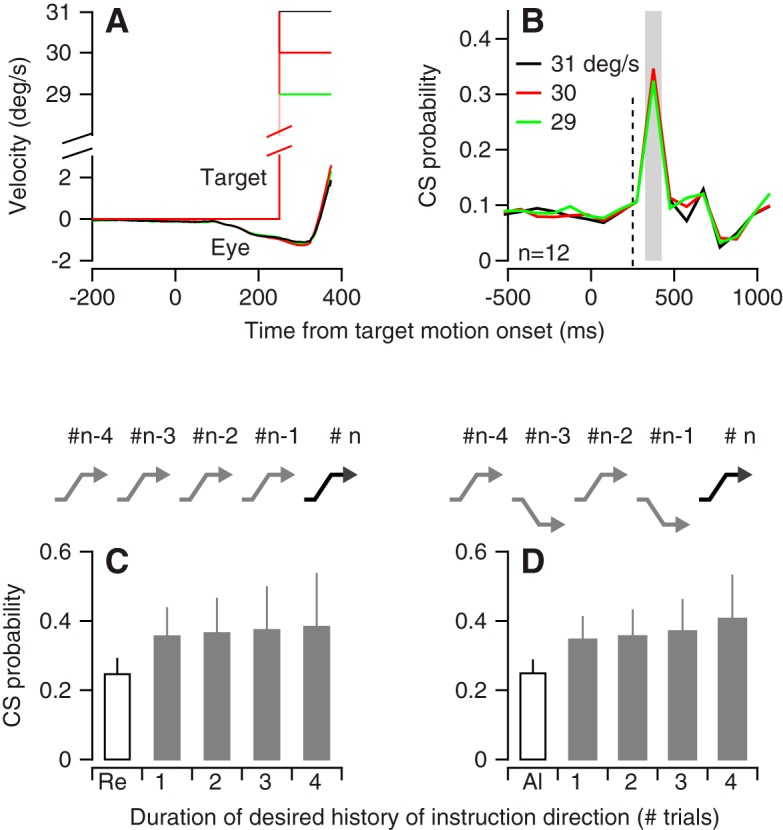
Controls showing that effect of learning paradigm depends on broader context of sequence of learning instructions. ***A***, Experimental design for delivering instructions with target speeds of 29, 30, or 31 deg/s. ***B***, CS probability versus time for target motion at different speeds. In ***A*** and ***B***, traces of different colors show data for different target speeds. ***C***, ***D***, CS probability as a function of the history of direction of instruction for the prior four trials. In ***C***, we selected from the random paradigm sequences of trials that repeat the off-direction target motion for up to four trials. Bar labeled Re shows CS probability in the repeated paradigm, and bars labeled with numbers show CS probability in the random-direction paradigm after sequences of one, two, three, or four instructions of the same direction. In ***D***, we performed the same analysis for sequences of alternating direction instructions during the random direction paradigm.

We performed two control analyses to eliminate artifactual explanations for the data in Fig. 3. First, the differing progress of eye movement learning in the different paradigms causes small, 1–2 deg/s differences across paradigms in the image motion across the retina caused by instructive changes in target direction. To test whether these tiny differences in the sensory stimuli alter CS responses, we introduced target motions in the off-direction for simple-spike responses at speeds of 29, 30, and 31 deg/s. Across the 12 Purkinje cells we tested, CS probability did not vary systematically in relation to target speed within this small range (Fig. 4*A*, *B*) and consistently reached a peak that was very close to 0.4. Second, we showed that CS probability was not modulated simply by recent history (Fig. 4*C*, *D*). To do so, we found sequences of trials from the random-direction block that mimicked the alternating or repeated order of instructive target motion. We then measured the probability of a CS in the *n*th trial contingent on whether the previous history followed the repeated or alternating sequence for one, two, three, or four trials. When embedded in the random-direction paradigm, sequences of up to four previous repeated (Fig. 4*C*) or alternating (Fig. 4*D*) directions of instructive target motion did not reduce the probability of a CS, which remained close to 0.4. Thus, we conclude that the effect of the learning paradigm on the probability of CS responses reflects modulation of climbing-fiber activity over a longer time scale than just a few trials.

### Comparison of potentiation and depression for on- versus off-direction learning

The learning curves for simple-spike firing on the trials that followed on- versus off-direction learning showed interesting differences across learning paradigms, suggesting two independent processes with separate mechanisms. Here, we measured simple-spike firing rate in the interval from 100 ms before to 50 ms after the onset of the instructive change in target direction, subtracted the baseline from before the learning session, and averaged across groups of 10 trials, separately for test trials that followed on-direction versus off-direction instructions.

**Figure 5.**
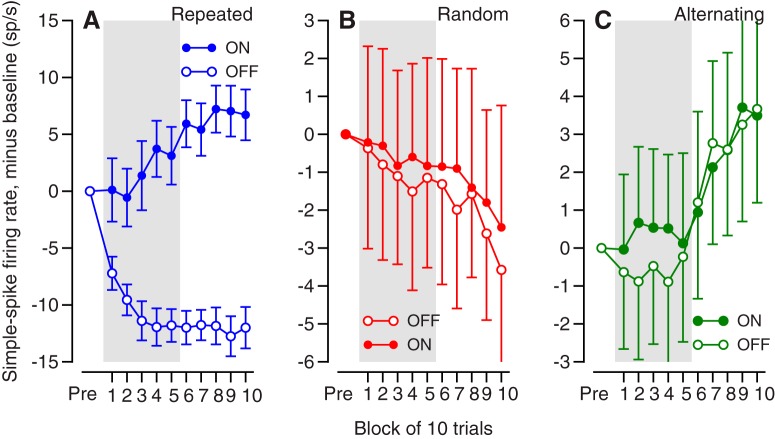
Comparison of time courses of simple-spike learning in the on- and off-directions for the repeated, random, and alternating-direction paradigms. Each symbol plots the simple-spike firing in the interval from 100 ms before to 50 ms after the onset of an instructive change in target direction, with the baseline simple-spike firing rate subtracted. Filled and open symbols show the firing rate in trials that followed and on-direction versus and off-direction instruction. Error bars show SEMs across the 34 Purkinje cells in the sample. Gray shading indicates the first 50 trials in each block.

In the repeated-direction paradigm (Fig. 5*A*), as we have shown before (e.g., Fig. 6 of Yang and Lisberger, 2014b), the neural learning curves for simple-spike firing rate were not symmetrical for on- versus off-direction learning. For the 34 Purkinje cells in this sample, depression more or less reached an asymptote within 30 trials. In contrast, potentiation started more slowly and proceeded steadily through at least the first 80 trials.

In the random-direction paradigm (Fig. 5*B*), in which single-trial depression persists throughout a learning block and is larger than single-trial potentiation (Fig. 1*C*), depression accumulated throughout a 200-trial block of trials. In the interval from 100 ms before to 50 ms after the instructive change in target direction, simple-spike firing rate was always smaller in the trial after on off-direction instruction, but gradually became increasingly negative (relative to the prelearning baseline) throughout the learning block for both directions of instruction. Paired *t* tests on the averages within 10-trial bins for the 34 Purkinje cells revealed statistically significant differences between on- and off-direction learning for bins 5–10 (*p* = 0.016) but not bins 1–4 (*p* = 0.53).

In the alternating-direction paradigm (Fig. 5*C*), simple-spike firing was positive or negative for on- versus off-direction learning for the first 40 trials in each direction. Then, as complex-spike duration and probability decreased and trial-over-trial depression decreased, the simple-spike firing rates became equal for on- versus off-direction. Firing rate also started to increase steadily, as if potentiation now was gradually dominating the weakened trial-over-trial depression. Paired *t* tests on the averages within 10-trial bins for the 34 Purkinje cells revealed statistically significant differences between on- and off-direction learning for bins 1–4 (*p* = 0.009) but not bins 5–10 (*p* = 0.92).

The progression of potentiation for on-direction instructions and depression for off-direction instructions suggests that these opposing mechanisms of plasticity follow different dynamics and are differentially affected by the sequence and predictability of instruction directions. Potentiation seems to be more timid, but also more persistent and inexorable, over longer sequences of learning trials. Thus, our data are consistent with ideas about the contribution of multiple forms of plasticity to cerebellar motor learning, including both depression and potentiation of the parallel fiber to Purkinje cell synapse (Hansel et al. 2001; Schonewille et al. 2010; Carey 2011; Gao et al. 2012; De Zeeuw and Ten Brinke, 2015; Gutierrez-Castellanos et al. 2017).

### Data analyses to constrain a model of single-trial learning

Our final goal at the end of this article is to develop a computer model that explains many details of a complex data set about single-trial learning. To constrain the model, we next evaluate additional features of the statistics of CS probability and duration during the random-direction learning paradigm. In particular, we ask about the correlation between the duration and probability of a CS and about the possibility of coordinated slow fluctuations of CS duration across a block of learning trials.

**Figure 6.**
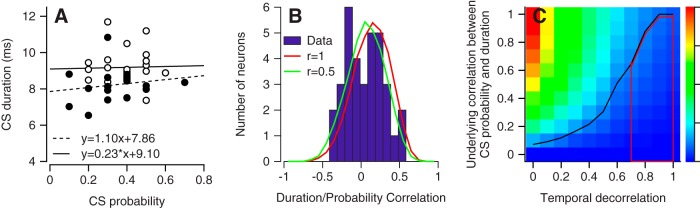
Low correlation between CS probability and duration measured in bins of 10 trials. ***A***, Graph plots CS duration versus probability. Open and filled symbols show data from two Purkinje cells, and each symbol shows measurements for a single bin of 10 trials. Lines were obtained by regression analysis. ***B***, Distribution of correlation between probability and duration of CSs. Blue bars show data from our sample of Purkinje cells. Red and green traces show predictions of the model described later in the paper, when the underlying correlation between CS probability was 1 (red) or 0.5 (green) and the degree of temporal decorrelation was set at 0.8. ***C***, Full grid of measured correlation between CS duration and probability in 10-trial bins as a function of the parameters of the model: underlying correlation between duration and probability and temporal correlation in CS duration value (temporal correlation in this graph is equal to 1 minus the temporal decorrelation parameter of the model). Black curve shows the values that yielded a measured correlation between CS duration and probability equal to 0.1 and defines the upper limit of biologically compatible values of the model parameters. Red traces outline the region that is compatible with the measured temporal correlation in CS duration and delimits the pixels below that are compatible with all the data.

To evaluate correlations between CS probability and duration, we first performed the obvious data analysis. We broke the sequence of trial pairs from the random paradigm into bins of 10 pairs with an off-direction instruction and measured the mean duration and the probability of a CS response. Plotting probability versus duration (Fig. 6*A*) revealed relationships that varied in strength from neuron to neuron. As a general rule, the correlation between CS probability and CS duration was weak for almost all neurons, and sometimes was even negative (Fig. 6*B*). The population was distributed fairly evenly around a mean correlation of 0.05.

To interpret Fig. 6*B*, we need to distinguish between (a) the underlying correlation of CS duration and probability and (b) the measured correlation between duration and probability. The underlying correlation determines whether an individual CS occurs and what its duration will be. Because we cannot evaluate probability on single trials, our data analysis does not have direct access to the underlying correlation. The measured correlation is a product of data analysis, which we performed by binning the data into groups of 10 trials. Averaging across 10 trials has the potential to dilute any correlations in the underlying probability and duration so that the measured correlations become quite small (e.g., Fig. 6*B*). However, it is still plausible that on any given trial, the underlying probability of a CS is strongly correlated with its duration.

Simulations of the model described in Materials and Methods verified that the obvious approach of correlating probability and duration might not reveal high correlations even if the underlying correlation is strong. Instead, the measured correlation between probability and duration also depends on the strength of any slow fluctuations in CS duration across a learning block (temporal correlations). We contrived our model so that we could control independently the temporal correlations in CS duration and the underlying probability of correlation between CS duration and probability. If CS duration has weak temporal correlations, then the analysis in Fig. 6 would measure very little correlation between CS duration and probability even if their underlying correlation were close to 1 for each CS response. For example, the green and red curves in Fig. 6*B* show the distributions of measured correlations between CS duration and probability predicted by the model when the correlation between duration and probability for individual CS responses was 0.5 and 1, and the temporal correlation of CS duration was quite low (decorrelation value in the model of 0.8).

The powerful impact of the temporal correlation of CS duration on the measured correlation between duration and probability is evident in Fig. 6*C*. Here, we systematically created known correlations between the probability and duration of individual CS responses and used the approach described in Materials and Methods to control independently the degree of temporal correlation. When temporal correlation was maximal (column of colored cells at 1 on *x*-axis, Fig. 6*C*), the measured correlation of CS duration and probability in 10 trial bins was almost equal to the underlying correlation (values on *y*-axis). As we decorrelated CS duration, moving to the right on the *x*-axis, the measured correlation between CS duration and probability declined toward zero. Any of the combinations of underlying correlation and temporal correlation below the black curve yield measured correlations between CS duration and probability that are <0.1, and therefore are compatible with the data in Fig. 6*B*.

**Figure 7.**
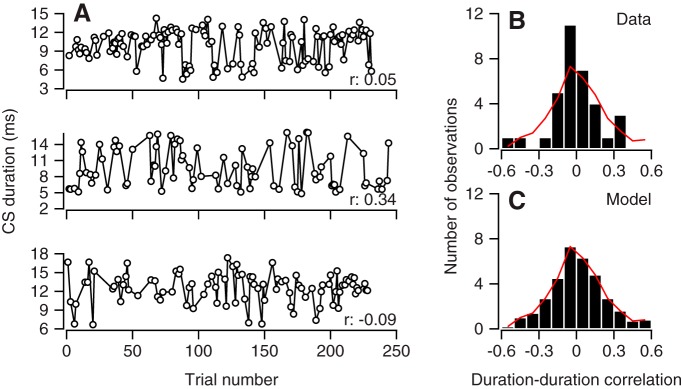
Absence of temporal correlation in duration of CS responses. ***A***, Duration of all CS responses in off-direction learning trials during a random direction learning block for three representative Purkinje cells. ***B***, Distribution of correlations between durations of CS responses in pairs of successive off-direction learning trials. ***C***, Distribution of correlations between durations of CS responses in randomly chosen pairs of trials. Number of observations is divided by 10, because we performed this analysis 10 times for each Purkinje cell, selecting the same number of pairs of trials as we had pairs of consecutive CS responses for that cell. The red curve in ***B*** and ***C*** reproduces the control distribution in ***C***.

We next evaluated temporal correlations of CS duration directly. Fig. 7*A* shows the sequence of CS durations in off-direction learning trials across random-direction learning blocks for three example Purkinje cells. Each cell shows some runs of similar CS durations, suggesting temporal correlation, and many runs of oscillation of CS duration, suggesting a lack of temporal correlation. To quantify temporal correlation, we extracted the CS durations on all pairs of consecutive trials with a CS response and calculated the correlation coefficient. High correlations between paired CS durations would suggest temporal correlations, whereas small or zero correlations between paired CS durations would suggest an absence of temporal correlations. For the three Purkinje cells in Fig. 7*A*, the correlations between successive CS durations were 0.05, 0.34, and –0.09. Across 32 qualifying Purkinje cells based on having >9 pairs of CS responses in consecutive trials (Fig. 7*B*), the temporal correlations averaged 0.03. For the repeated and alternating-direction paradigms, the mean correlations were 0.12 and 0.22 for *n* = 15 and 10 qualifying Purkinje cells. The correlations between the durations of successive CS responses were slightly larger than those between the durations of pairs of trials made up of randomly chosen CS responses (Fig. 7*C*, red curves in Figs. 7*B* and *C*, mean *r* = –0.03, 0.12, and –0.05 for random, repeated, and alternating-direction paradigms). We conclude that there is at best a small temporal correlation in CS duration. We are able to summarize the possible states of the system we studied as the area within the red polygon in Fig. 6*C*. This polygon is compatible with our data on measured correlation of CS duration and probability (Fig. 6*B*) and temporal correlation (Fig. 7*B*).

**Figure 8.**
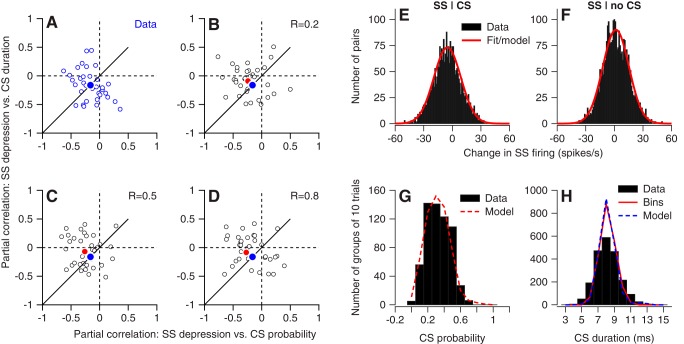
Agreement between statistics of model and data. ***A***, Structure of the relationship between partial correlations trial-over-trial changes in simple-spike firing with CS probability on the *x*-axis and duration on the *y*-axis. Symbols show different neurons. ***B–D***, The same analysis as in ***A***, but for the computational model with the underlying correlation of duration and probability set to 0.2, 0.5, or 0.8 and the temporal decorrelation set to 0.8. Blue and red filled symbols show the averages for the data and the model, respectively. ***E***, ***F***, Distributions of trial-over-trial changes in simple-spike firing, contingent on the presence (***E***) or absence (***F***) of a CS response to the instructive change in target direction on the first trial of the pair. Black histograms show the data across all Purkinje cells, and the red curve shows the best-fitting normal distribution, also used in the model. ***G***, Distribution of CS probability. Black histogram shows analysis of the data from all Purkinje cells in bins of 10 trials, and the red dashed curve shows the same analysis of the model Purkinje cells. ***H***, Distribution of CS durations. Histogram bars show the data based on single trials, continuous blue and dashed red curves show results for the data and the model, based on averaging across bins of 10 trials.

Finally, we assessed a second-order statistical feature of our data, namely the relationship between the separate effects of CS duration and probability on trial-over-trial depression of simple-spike firing rate in our data. For each Purkinje cell in our sample, we selected the pairs of trials with off-direction instructions in the random direction block and divided them into groups of 10 pairs. Then, we used the Matlab routine “partialcorr” to calculate the partial correlation between each of the two properties of CS responses (probability or duration) and trial-over-trial changes in simple-spike firing while controlling for the other property. A plot of the partial correlation of simple-spike depression with CS duration versus that for CS probability reveals a wide range of values in different neurons (Fig. 8*A*, open symbols). Two features are notable. First, some neurons showed larger correlations with CS duration, some with CS probability, and some with both. Second, the neurons plotted in quadrants II, III, and IV, but not in quadrant I.

### A computational model of single-trial learning

We regard the cell-by-cell variation in Fig. 8*A* as emblematic of the competing effects of legitimate variation related to trial-by-trial variation in neural responses, and structure related to the underlying mechanisms driving the average behavior of the system. In modeling single-trial learning, our goal was to show that we fully understand the system by capturing the structure, the variation, and the correlations in the data. That is, we created a model that included the variation in the parameters of CS and simple-spike responses, and we asked whether the model could reproduce both the structure and the variation in Fig. 8*A*. The model simulated responses for sequences of learning trials in the random-direction paradigm, but did not include the time in milliseconds within each trial. Thus, we made no effort to mimic the reliability/variability of CS timing in response to instructive changes in target direction.

The model incorporated the following mechanisms:

- CS probability and durations had distributions that matched our data (Fig. 8*G*, *H*).
- If a model Purkinje cell receives an input from its climbing fiber on a given trial, then its simple-spike firing rate on the subsequent trial is reduced by 5.5 spikes/s on average through CS-linked trial-over-trial plasticity. If the neuron fails to emit a CS on a given trial, then its simple-spike firing rate is increased by 1.6 spikes/s on average in the subsequent trial.
- The variations in the trial-over-trial depression or potentiation of model simple-spike firing were based on and mimicked the distributions in our data (Fig. 8*E*, *F*).
- Only an off-direction instruction can evoke a CS response and, in accordance with our data, evoked one or zero CS responses. On-direction instructions never evoked CS responses (Medina and Lisberger, 2008). Therefore we did not include ON-learning trials in our simulations.
- The CS-linked trial-over-trial depression in simple-spike firing varied systematically as a function of CS duration (Yang and Lisberger, 2014a).
- CS duration has low temporal correlations (decorrelation parameter of 0.8), within the bounds defined by our data (Fig. 6*C*).
- As shown in our prior article (Yang and Lisberger, 2010), trial-over-trial depression or potentiation of simple-spike firing rate was forgotten within two trials.

We created a population of 1000 Purkinje cells and ran the model for 200 off-direction learning trials. Using equations given in Materials and Methods, we varied the parameters of the model, simulated the trial-over-trial changes of simple spike firing across the population, and analyzed the simulated data exactly as we had analyzed the real data to obtain Figs. 8*A*.

The results from simulation of the model mimicked the data and did not depend in any obvious way on the underlying correlation between CS probability and duration (Figs. 8*B–D*). The means of the partial correlations are negative, and the different model Purkinje cells plot in quadrants II, III, and IV of the graph.

We created the model analyzed here with the hope that it would allow us to use the data in Fig. 8*A* to constrain the underlying correlation between the probability and duration of CS responses. That it did not was a disappointment. Still, the model does provide a lesser but still important contribution. It shows that we have a really complete understanding of single-trial plasticity of simple-spike responses, because we can create a model that is based entirely on our measurements from Purkinje cells and reproduce even the second-order statistics represented by the structure of Fig. 8*A*.

## Discussion

Our prior articles have shown that (1) the direction of pursuit is subject to learning (Medina et al. 2005), (2) behavioral learning can occur after a single behavioral trial (Yang and Lisberger, 2010), (3) there is a neural expression of single-trial learning in the simple-spike firing of Purkinje cells in the floccular complex (Medina and Lisberger, 2008; Yang and Lisberger, 2014b), (4) single-trial neural learning is tightly linked to the occurrence of a climbing-fiber input on the prior trial (Medina and Lisberger, 2008; Yang and Lisberger, 2013), and (5) the magnitude of both behavioral and neural learning on any given trial is related to the duration of the CS caused by the climbing fiber on the prior trial (Yang and Lisberger, 2014a). Now, we outline additional correlations between the size of neural learning and the properties of the CS responses to instructions for learning. In addition, we demonstrate a model of neural learning that accounts for the statistics of our observations in some detail.

The system and cerebellar structure we study, smooth-pursuit eye movements and the floccular complex, are ideally suited for analysis of the neural basis for motor learning because we know the anatomic and physiologic relationship between Purkinje cells in the floccular complex and extraocular motoneurons. Anatomically, floccular Purkinje cells inhibit their target neurons in the vestibular nucleus (Lisberger et al. 1994), and these in turn inhibit motoneurons and presumably internuclear neurons in the abducens nucleus (Highstein, 1973). Because of the double inhibitory connection, increases in the simple-spike firing of floccular Purkinje cells should excite ipsilateral abducens neurons and cause ipsiversive eye movement. The physiologic relationship between Purkinje cell activity and eye movement during baseline pursuit fits into this anatomic framework: increases and decreases in simple-spike firing rate are associated with ipsiversive versus contraversive pursuit (Krauzlis and Lisberger, 1996). The arrangement seems to be analogous for Purkinje cells that have vertical (downward) preferred directions (Partsalis et al. 1995). The physiologic relationship also holds for pursuit learning (Medina and Lisberger, 2008, Yang and Lisberger, 2013): on-direction learning in the repeated-direction paradigm leads to eye movements in the on-direction and increases in simple-spike firing rate (potentiation), whereas off-direction learning leads to eye movements in the off-direction and decreases in simple-spike firing rate (depression). The directions of the single-trial neural plasticity studied in the present article fit into this framework as well.

In our first description of single-trial learning in pursuit (Yang and Lisberger, 2010), we noted that the magnitude of trial-over-trial behavioral learning depended on the context of a given pair of trials. Single-trial learning was largest when we randomized the order of the instruction directions, weaker (but in the direction specified by the instruction) when the direction of the instructions alternated, and asymptotically relatively weak when we repeated the same instruction direction 100 times.

In the present article, we show that both the probability and duration of CS responses in floccular Purkinje cells track the strength of behavioral learning. Over the course of 100 repetitions of instructions in the same or alternating directions, both the probability and duration of CS responses decrease in good agreement with the time course of decreases in the magnitude of trial-over-trial neural and behavioral learning. In contrast, the CS properties remain constant at a high level of probability and duration, along with trial-over-trial behavioral and neural learning, when we randomize the order of directions. Importantly, CS duration does not show coordinated slow fluctuations (a.k.a. temporal correlations) in the random direction paradigm, suggesting that external modulation, rather than intrinsic properties of the olivo-cerebellar system, causes the highly coordinated gradual decline in CS duration in the repeated and alternating learning paradigms. Our control experiments show that the effects on CS probability in the repeated and alternating paradigms are due to the broader context set by the sequence of instructions, not to either the recent history of instruction directions or minor trial-to-trial variations in the retinal image motion created by the instructive change in target direction.

We suggest that the broader learning context set by the sequence of learning directions leads to descending modulation of complex-spike responses (Najafi and Medina, 2013), and in turn to more or less learning on a single-trial basis. We imagine that this modulatory capacity could be the basis for voluntary control of the potential for learning under conditions when learning is a good versus a bad idea. The fact that the direction of behavioral learning is guided by the direction of the instruction on the prior trial, even when the instruction directions alternate reliably over 200 trials, suggests that the modulation of CS properties occurs at a subconscious level, at least under the conditions of our experiments.

Our experiments do not address whether modulation of CS duration occurs in the inferior olive, where it might regulate the duration of the burst that travels up the climbing-fiber axon, or in the cerebellar cortex, where the state of the Purkinje cell membrane might regulate the duration of the CS caused by a given burst. Mossy-fiber inputs cause Purkinje cell depolarization and changes in the state of the Purkinje cell membrane (Najafi et al. 2014) that might affect CS duration, at least under some conditions (e.g., Warnaar et al. 2015; Burroughs et al. 2017). In our prior article (Yang and Lisberger, 2014a), we argued that the modulation of CS duration occurred in the inferior olive because of a lack of correlation between CS duration and simple-spike firing rate, the latter an indicator of the state of the Purkinje cell membrane. If our analysis had been able to constrain the degree of underlying correlation of CS probability and duration, we might have been able to shed some light on the site of the modulations.

Still, it is appealing to think that the level of excitability across the inferior olive is a key common variable, and that excitability fluctuates from trial to trial (Mathy et al. 2009). A higher level of excitability might increase the chance of a response to a given sensory input and at the same time bias the response toward a longer burst of output spikes that would lead to a CS of longer duration (Maruta et al. 2007; Bazzigaluppi et al. 2012) and larger plasticity (Rasmussen et al. 2013; Yang and Lisberger, 2014a). Given that both the probability of a climbing-fiber event and its duration alter the amount of neural learning in the cerebellum (Medina and Lisberger, 2008; Yang and Lisberger, 2014a), joint modulation of the two parameters seems to be a sensible way for descending control to modulate the amount of cerebellar learning depending on context.

Finally, we have presented a simple computational model that is based on our analysis of the statistics of CS probability and duration and of single-trial learning in simple-spike responses. The model reproduces the partial correlations between the trial-over-trial changes in simple-spike firing and both CS duration and probability. Given the features we built into the model, it is not surprising that it reproduces our data qualitatively. The fact that it does so quantitatively suggests that the data in this study provide a fairly complete account of the factors that mediate single-trial learning linked to climbing-fiber inputs to the cerebellar cortex. We suspect that the mechanism we call single-trial learning has broad implications, given that it now has been uncovered in smooth-pursuit eye movements (Medina and Lisberger, 2008), the vestibulo-ocular reflex (Kimpo et al. 2014), and classic conditioning of the eyelid response (Ten Brinke et al. 2015; Khilkevich et al. 2016).

## Synthesis

Reviewing Editor: Christian Hansel, University of Chicago Decisions are customarily a result of the Reviewing Editor and the peer reviewers coming together and discussing their recommendations until a consensus is reached. When revisions are invited, a fact-based synthesis statement explaining their decision and outlining what is needed to prepare a revision will be listed below. The following reviewer(s) agreed to reveal their identity: Samuel Wang, Chris I. De Zeeuw.

In this interesting paper, the authors nicely describe the impact of CSs on trial-by-trial learning in a visually guided motor task. The paper is well written and nicely and clearly illustrated, and as always with papers from the Lisberger lab, it is a great pleasure to read, independent from the fact whether the reader is an insider or outsider. The authors follow up on several previous studies from the Lisberger group, notably a study by Yang and Lisberger that examined the role of complex spike (CS) duration on trial-to-trial learning. Here the authors broaden their attention to CS probability as well.

- Introduction: Please bring up the possibility that potentation at the PF-PC synapse might also contribute to a learning process in the vestibulocerebellum, particularly during the absence of climbing fiber activity (see e.g. Gutierrez et al., 2017 Neuron; De Zeeuw and Ten Brinke, 2015 CSHP; Schonewille et al., 2010 Neuron).

- Methods: Please describe in greater detail how the spatial tuning and characterization of the complex spikes and simple spikes of the Purkinje cells were done.

- It is nice to include the model, but please provide a more detailed motivation for the choice of distributions in the model for the generation of complex spikes. Now, it seems a bit arbitrary that the complex spikes are generated by deductions from a normal distribution, which is thresholded by another draw from a uniform distribution. Likewise, please explain why the simple spikes are assumed to be normally distributed. Are they actually normally distributed?

- Results: Separation between “CS-frequent ” vs. “CS-infrequent ” Purkinje cells (lines 210-224) based upon previously published criteria. Can the authors indicate for the current data set, how many Purkinje cells were CS-frequent/-infrequent? Did the “CS-infrequent ” Purkinje cells have comparable simple-spike modulation compared to the CS-frequent Purkinje cells? Were all cells included in this study “CS-frequent ”? If so, how does this relate to the average fractions of complex-spikes per trial of 0.24 and 0.25, respectively (line 284)? Maybe the “CS-infrequent ” Purkinje cells are prone for potentiation and maybe these cells are actually relevant for the behavioral outcome. If not, this would also be an interesting conclusion. In other words, providing this extra analyses (see also below), would really strengthen this manuscript, independent from a negative or positive outcome.

- Lines 268-270: “...the monkey knew... ”: To what extent was the cognitive understanding of the trial structure by the monkey tested?

- Lines 270-272: “algebraic identities make the learning curve for ON-direction instructions identical to that for OFF-direction instructions, but with a positive sign. ” Is this true? ON-direction instructions typically lack a complex-spike. Does that not have an effect? In other words, unless I overlook something, ON-direction instructions are not merely the mirror image of OFF-direction instructions. compare this to the remark at lines 432-434: “Only an OFF-direction instruction can evoke a CS response. ON-direction instructions never evoked CS responses (Medina and Lisberger, 2008). Therefore, we do not need to include ON-learning trials in our simulations. ” Does this not question the requirement of CS firing for motor learning? Since we know that the absence of CSs can enhance SS activity and modulation (e.g. De Zeeuw and Ten Brinke, 2015 CSPH; Gao et al., 2012 Nat Neur Rev), possibly these other ON-direction cells are actually very interesting to look at and compare their behavioral impact with the cells analysed so far, just like what is stated above for the CS-infrequent ” Purkinje cells.

- In the statistics for Fig. 2e and f, a paired T-test is used. However, no mention is made of a correction for multiple comparisons, and thus possibly inflating the probability of false positives. If not done, please redo the statistics while controlling for multiple comparisons. E.g. through a repeated measures ANOVA, or applying a Bonferroni correction or so.

- It is stated that the behavioral learning correlates with the neural learning. From looking at the figures, I agree. Please provide some statistics to strengthen this conclusion.

- With regard to the relations tested for CS duration, would it make sense to also test the number of spikelets rather than the absolute duration? Hausser and colleagues showed that the number of spikelets might correlate to the level of LTD and LTP (Mathy et al., 2014; Neuron). This aspect should at least be discussed, together with a discussion of types of plasticity that modify the complex spike waveform (# spikelets and/or duration), in Hansel and Linden 2000 (Neuron) and Ohtsuki and colleagues (2012, Neuron).

- Discussion: Please discuss here why SS suppression (or depression) is more likely to do the job than the SS potentiation? Does it fit with the network connectivity in the vestibular nuclei? How does less inhibition in PC activity lead to the proper eye movements, given the inhibitory connections involved in the downstream circuitry? Is rebound activity a requirement in this respect or not?

- With regard to the intrinsic mechanisms underlying CS duration, please also discuss the paper by Warnaar and colleagues (2015, Frontiers) and Burroughs and colleagues (2016, J Physiol).

- Line 518, when discussing the trial-by-trial learning and analysis of eyeblink data, please refer also to Ten Brinke et al., 2015 (Cell Reports), who were the first to do so. In this respect, it would also be interesting to make a more thorough comparison between smooth pursuit and eyeblink learning, during both of which SS suppression may be necessary. Indeed, here rebound activity following less inhibitory drive from the PCs might contribute to the amplitude of the CRs (De Zeeuw and Ten Brinke, 2015).

- Conclusions: For the model a decorrelation of 0.8 is mentioned for temporal correlation, while earlier 0.6 was mentioned in the text. Is this an observed value, or a parameter set for the model as mentioned in the text of Fig. 5? Please clarify.

- The last claim about the model 'Trial-over-trial depression or potentiation of simple-spike firing rate was forgotten within two trails' (line 439) was not really demonstrated or substantiated by statistics as far as I can see. Please do so.

- The implications of the model are somewhat unclear. The authors show that they can obtain a similar variation in changes in trial-by-trial SS firing rates, given a CS or no CS in the preceding trial, compared to measured data. However, this is an aggregate measure, whereas the authors claim to have “a clear understanding of learning in floccular Purkinje cells on the basis of their response properties ”. The single-trial accuracy of the model is not demonstrated, I think.

- It would also be nice to see the timing of complex spikes presented in the model, especially since in the first figure it was shown that complex spikes are quite precisely timed. In addition, the model is constrained to 0 or 1 complex spike per trial, whereas in reality multiple complex spikes may be possible.

- Figures: The way Fig. 1B is presented now is not very informative. This reviewer feels it would be really worth it to present a complete scheme for smooth pursuit control, all the way from the olivocerebellar system up to the eye muscles. It would be nice to highlight in such a scheme, why SS suppression and/or potentiation actually would make sense in terms of learning direction and network connectivity, including the inhibitory or excitatory nature of the neurotransmission.

- Fig. 1D: please indicate type of trials also in figure legend.

- Fig. 2B: What is the unit on the y-axis? And how does this relate to Fig. 1D, where absolute velocities of 12 deg/s were reached (cf. -2 in Figure 2B).

- Fig. 5: Please give unit in y-axis (ms).

- In Fig. 6, the authors argue that there is little or no temporal correlation of the CS duration during a block of random direction learning. To what extent is this conclusion different from the absence of change in the CS duration as illustrated in Figure 3K? And what about the temporal correlations during repeated / alternating blocks?

- In the text there is a reference (line 401) to a red curve in Fig. 6c; however, a red curve is present in Fig. 6b, but not Fig. 6c.

- In Fig. 7a-d, the x and y-axis have the same title: Partial correlation: SS duration vs CS duration; I assume that one on the x-axis should be related to CS probability.

- Line 30, “A burst of action potentials ”: Is this demonstrated? I thought olivary neurons could also fire a single AP. If possible, add citations that mention how many times an olivary neuron or climbing fiber fires. It might be necessary to moderate the statement.

- Line 50, “...depending on context. ” It might be worth mentioning that CS millisecond synchrony provides an additional mechanism by which instructive strength might be modulated. This synchrony is worth mentioning because it is likely to vary with probability on a trial-by-trial basis, and because it opens the possibility of other learning sites that depend on multi-Purkinje-cell CS activity, notably the deep nuclei.

- Line 167, “determines ”: maybe change to “r equals ”?

- Line 171: this expression simplifies to d/4 - 1.

- Lines 494-495, “the state of the Purkinje cell membrane ” This is given somewhat short shrift. It might be a good place for a brief mention of Najafi/Giovannucci's papers (Najafi and colleagues, 2014 Cell Reports) suggesting that mossy-fiber pathways could cause Purkinje-cell depolarization.
